# Influence of sustained mild dehydration on thermoregulatory and cognitive functions during prolonged moderate exercise

**DOI:** 10.1007/s00421-024-05548-6

**Published:** 2024-07-10

**Authors:** Hironori Watanabe, Yuma Kadokura, Taisuke Sugi, Kiyoshi Saito, Kei Nagashima

**Affiliations:** 1 Institute for Energy and Environmental System, Sustainable Energy & Environmental Society Open Innovation Research Organization, Waseda University, 3-4-1 Okubo, Shinjuku-ku, Tokyo, 1698555 Japan; 2 Advanced Research Center for Human Sciences, Waseda University, 2-579-15 Mikajima, Tokorozawa, Saitama 3591192 Japan; 3 Body Temperature and Fluid Laboratory, Faculty of Human Sciences, Waseda University, 2-579-15 Mikajima, Tokorozawa, Saitama 3591192 Japan; 4 Infrastructure Systems Research & Development Center, Toshiba Infrastructure Systems & Solutions Corporation, Kawasaki, Kanagawa 2129595 Japan; 5 Department of Applied Mechanics and Aerospace Engineering, School of Fundamental Science and Engineering, Waseda University, 3-4-1 Okubo, Shinjuku-ku, Tokyo, 1698555 Japan

**Keywords:** Hyperosmolality, Hypovolemia, Euhydration, Thermoregulation, Heat stress, Water-intake restriction

## Abstract

**Purpose:**

The current study investigated whether sustained mild dehydration affects thermoregulatory function and cognitive performance during prolonged exercise.

**Methods:**

Twelve young adults performed a test consisting of three sets of 20-min exercise with 2-min intervals under euhydrated (control, CON) and mildly dehydrated conditions (MDEH) at an ambient temperature of 30 °C and 60% relative humidity. MDEH was established by restricting water intake for 24 h, resulting in urine specific gravity of ≥ 1.020. Heart rate (HR), mean arterial blood pressure (MAP), skin blood flow (SkBF), sweat rate (SR) on the chest and forearm, and ear canal and mean skin surface temperatures (T_ear_ and mean T_skin_, respectively) were continuously recorded. For each exercise set, thermal and humid sensations and thermal discomfort were assessed using visual analog scales (VAS), and the rating of perceived exertion (RPE) was estimated. Cognitive performance on the Go/No-Go (easy) and incongruent Stroop (difficult) tasks was assessed before and after the test.

**Results:**

No differences were observed in HR, MAP, SkBF, SR, T_ear_, and mean T_skin_ between the CON and MDEH. Thermal and humidity sensations, thermal discomfort, and RPE were higher in MDEH than in CON. Moreover, response time to the Stroop task was prolonged in MDEH.

**Conclusion:**

These findings suggest that sustained mild dehydration does not affect autonomic thermoregulation during exercise. Augmented thermal perception and perceived exertion, which are necessary for behavioral thermoregulation, were noted; however, cognitive function may be attenuated under MDEH.

## Introduction

Maintaining adequate fluid intake is crucial for various vital functions in humans (Sawaka et al. 2005; Armstrong and Johnson 2018). Insufficient fluid intake to compensate for fluid loss due to urination, skin surface evaporation, respiration, and/or sweating results in dehydration (Kenefick and Cheuvront 2016; Epstein and Yanovich 2019). Dehydration often affects children (Bar-David et al. 2005; D’Anci et al. 2006) and the elderly (Suhr et al. 2004), whose ability to regulate fluid balance, including renal function, drinking behavior, and vascular control, is limited. However, dehydration is also observed in healthy adults.

Prolonged exercise and physical labor in hot environments are common stimuli that induce dehydration due to excess sweating in healthy adults. Such dehydration is often accompanied by a decrease in blood volume (hypovolemia) and an increase in plasma osmolality (hyperosmolality), factors that attenuate autonomic thermoregulatory responses, such as sweating and skin vasodilation (Sawka 1992). Garcia et al. (2022) hypothesized that dehydration may increase the risk of exertional heat stroke via hyperthermia. However, in a laboratory study that imposed hypohydration, participants ceased exercise due to exhaustion at a lower core body temperature than when hypohydrated (Sawka 1992). Therefore, in daily life, the risk of heat stroke due to dehydration may be limited to environments where water intake and/or protective behavior are unavailable. In contrast, an animal study showed that mice under hyperosmotic conditions exhibited attenuated escape behavior from heat and selected warmer places (Lin et al. 2012). In humans, hyperosmolality attenuates heat sensation (Tokizawa et al. 2016). Such changes in thermal sensation may enhance the risk of heat illness. However, there is little evidence to implicate dehydration as a mediator of heat illness and the role of behavioral responses in such conditions.

The influence of dehydration on thermoregulatory responses to heat has mostly been examined under experimental conditions of moderate-to-severe dehydration induced by exercise in hot environments with restricted water intake or by administration of diuretics (Garcia et al. 2022). However, healthy individuals often experience mild dehydration in their daily activities (Sawka et al. 2007), which is defined as a 1–2% loss of body weight with urine specific gravity (USG) of ≥ 1.020, indicating concentrated urine (Perry et al. 2016). Public surveys have also indicated that some groups of people who work and exercise in hot environments, such as factory workers, firefighters, military personnel, and athletes, have sustained mild dehydration due to inadequate rehydration (Brake and Bates 2003; Stover et al. 2006; Kenefick and Sawka 2007; Peacock et al. 2011). In addition, such dehydration is observed even at the onset of work and training (Brake and Bates 2003; Stover et al. 2006; Kenefick and Sawka 2007; Peacock et al. 2011). Plasma hyperosmolality is a strong factor attenuating autonomic responses to the heat, such as increases in skin blood flow and sweat rate (Tokizawa et al. 2010). However, Perry et al. reported that, during sustained mild dehydration, the level of plasma osmolality was maintained within a normal range (~ 290 mosmol/kg) (Perry et al. 2016). The impairment of thermoregulation due to such isotonic dehydration is limited to skin blood flow but not sweat rate (Ikegawa et al. 2011; Takamata 2012). The results may suggest that, in heat, the influence of sustained mild dehydration on autonomic thermoregulation is limited. However, it remains unclear if the sustained mild dehydration attenuates thermoregulation in heat and increases the risk of heat stroke.

Dehydration also affects brain function. Dehydration-induced electrolyte imbalance in body fluids affects the neurotransmitter systems involved in cognitive processing in the brain (Wilson and Morley 2003; Lieberman 2007; Adan 2012). Moreover, dehydration disturbs the function of the blood–brain barrier, increases vascular permeability, and decreases blood flow to certain brain regions (Maughan et al. 2007). Moreover, even sustained mild dehydration impairs cognitive function (Posada-Quintero et al. 2020). These results suggest that sustained mild dehydration blunts behavioral responses to heat, such as avoidance behavior and the decision to stop working or exercising, thereby increasing the risk of heat stroke. However, whether impaired cognitive function is related to thermoregulatory behavior and work and/or sports performance remains unclear.

This study aimed to investigate whether sustained mild dehydration adversely affects thermoregulatory function during moderate exercise in warm environments. Sustained mild dehydration was induced by water restriction overnight. In addition, we evaluated the thermoregulatory function from several viewpoints, including autonomic thermoregulation, thermal perception, and cognitive function related to exercise. Therefore, we examined (1) autonomic thermoregulatory responses of skin vasodilation and sweating, including changes in body temperature; (2) thermal perception of heat; (3) subjective evaluation of fatigue associated with exercise; and (4) cognitive performance in tasks.

## Methods

### Ethics statements

All experimental procedures and protocols conformed to the Declaration of Helsinki and were approved by the Human Research Ethics Committee of Waseda University (Approval Number: 2023-212). To avoid bias, participants were informed of the experimental procedures but were not informed of the aim and hypotheses. The participants believed that the experiment was conducted to examine the effects of two different hydration states on physiological and psychological variables. All participants provided informed consent before participating in the experiment. At the end of the experiment, the participants were debriefed on the study’s aim and requested not to share this information with other scheduled participants.

### Participants

Twelve right-handed young adults (nine male and three female adults; age, 22 ± 2 years; height, 166.6 ± 8.8 cm; body weight, 58.56 ± 9.68 kg; peak oxygen uptake [$\dot{V}$O_2peak_], 42.79 ± 7.11 mL/min/kg [mean ± standard deviation]) were recruited for this study. All participants were students in the same university who attended English classes, indicating that their study progress related to cognitive tasks was comparable. All participants were non-smokers, did not have any neuromuscular diseases, and did not take any medication. All participants engaged in recreational activities, such as at least 30 min of low- (e.g., walking) or moderate-intensity (e.g., jogging) aerobic exercise for 3–5 days per week. Moreover, considering their $\dot{V}$O_2peak_, they could be involved in performance level 2 in the classification of subject groups of sports science research (Decroix et al. 2016). In addition, to avoid confounding factors related to the intra- and inter-individual heat acclimation, this study was conducted between October (autumn) and December (winter) in Japan. Hence, all participants were classified as normal, healthy adults without heat acclimation.

### Experimental procedures

Participants visited the laboratory on three separate occasions, with a washout period of at least seven days between each visit. At the first visit, participants underwent an incremental exercise test on a treadmill to determine their $\dot{V}$O_2peak_ and the corresponding running speed. After the nude body weight was measured, the participants were instructed to change into a set of clothing, consisting of an underwear, T-shirt, gym shorts, and socks to wear during the experiment. The participants entered an artificially controlled environment chamber (ambient temperature, 25.0 °C; relative humidity [RH], 50.0%) (TBR-12H; Espec, Osaka, Japan) and were seated for at least 10 min. The participants then stood on the treadmill for 2 min before starting the test, consisting of walking at 3 km/h for 3 min and gradually increasing the speed by 0.1 km/h every 6 s until exhaustion. The breath-by-breath method was used to determine $\dot{V}$O_2peak_ using a respiratory metabolism device (AE-100i; Minato Medical Science, Osaka, Japan). After the test, the participants completed at least two Go/No-Go tasks (50 trials) and incongruent Stroop tasks (50 trials) to minimize learning effects. These tasks were conducted to assess the executive function related to attentional load, set-shifting, and inhibitory control among the cognitive functions in the second and third tests (Stroop 1935; Aron et al. 2007).

**Fig. 1:**
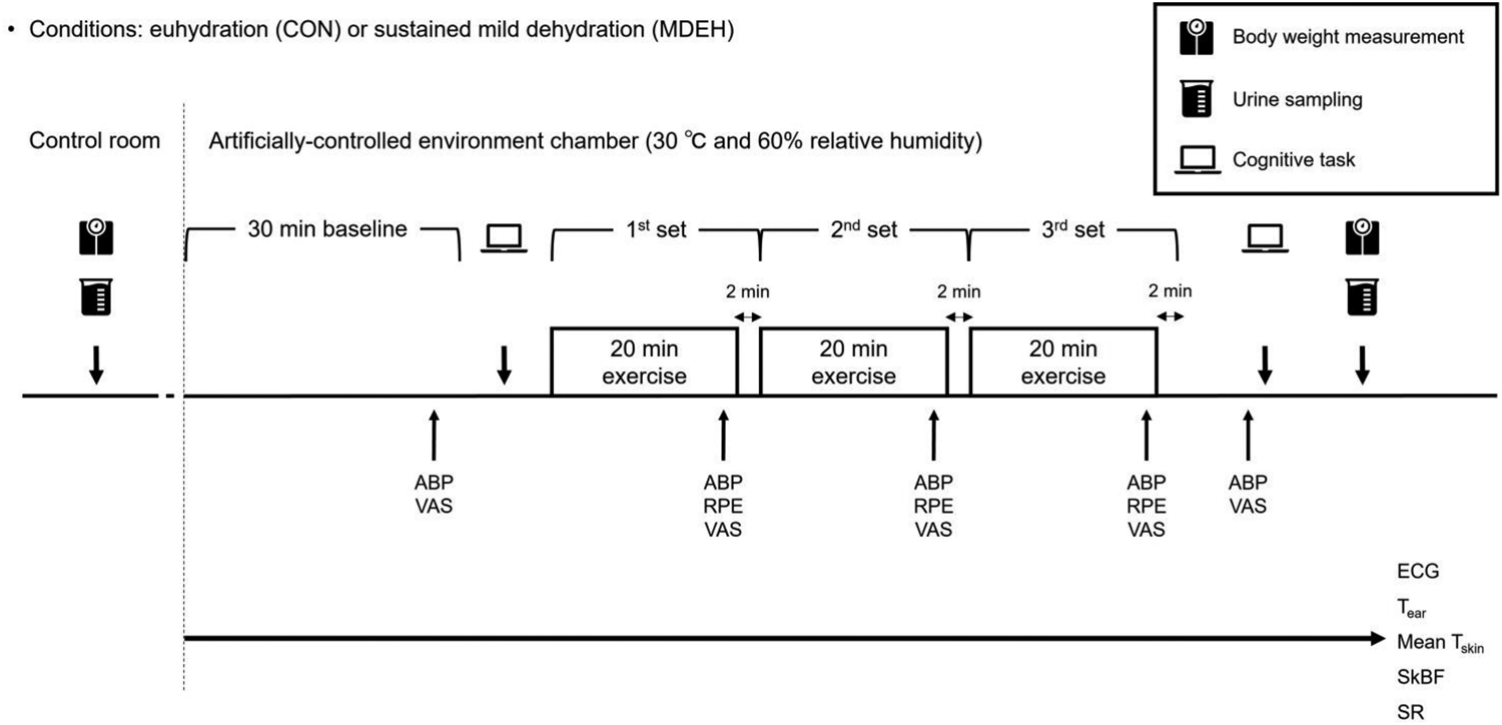
Illustration of the experimental protocol. *ABP* arterial blood pressure, *VAS* visual analog scale, *RPE* rating of perceived exertion, *ECG* electrocardiogram, *T*_*ear*_ ear canal temperature, *mean T*_*skin*_ mean skin surface temperature, *SkBF* skin blood flow, *SR* sweat rate

Figure 1 presents an overview of the experimental protocol during the second and third visits. The participants underwent exercise tests under either euhydrated (control [CON]) or sustained mild dehydrated conditions (MDEH) in a randomized and counterbalanced order. The tests were conducted at the same time of the day after the participants were instructed to abstain from consuming a meal (see Intervention) 3 h prior to the test and caffeinated beverages, alcohol, and strenuous exercise 24 h prior to the test. Upon arrival at the laboratory, a urine sample was collected, and the hydration state was assessed by measuring USG via refractometry (Atago Hand Refractometer; AstraZeneca, Osaka, Japan). CON was defined as USG ≤ 1.010, whereas MDEH was defined as USG ≥ 1.020 (Sawka et al. 2007; Perry et al. 2016). After nude body weight was measured, the participants wore the same clothing as the first visit and entered the artificially controlled environment chamber (ambient temperature, 30.0 °C with 60.0% RH; wet-bulb globe temperature [WBGT], 28.0 °C). Although the present study aimed to elucidate the effects of MDEH on thermoregulatory function leading to the increase in the risk of heat stroke, this environmental setting was selected based on the consideration that conducting the experiment in an excessively hot environment could lead to more pronounced effects on physiological indices (i.e., increased sweating), potentially masking the effects of MDEH. The participants were then seated for 30 min while the experimental equipment was fixed. Subsequently, they completed the Go/No-Go (50 trials) and incongruent Stroop tasks (50 trials). After a 5-min rest on the treadmill, the participants performed a 60-min moderate-intensity exercise test (40% $\dot{V}$O_2peak_), consisting of three sets of 20-min exercise, and each set was separated by a 2-min rest period. During the test, $\dot{V}$O_2_ was continuously monitored by the breath-by-breath method (AE-100i; Minato Medical Science, Osaka, Japan). Before and after the trial, thirst sensation was evaluated using a visual analog scale (VAS). During the test, subjective thermal and humid sensations, thermal comfort, and physical and psychological fatigue were evaluated by VAS 2 min before the end of the baseline and exercise sets. Only during exercise set, the rating of perceived exertion (RPE) was also assessed at the same timepoints. Subsequently, the participants were seated for 8 min and completed the VAS, Go/No-Go (50 trials), and incongruent Stroop tasks (50 trials). The verbal instructions for VAS and cognitive tasks were provided in the same manner for each condition to avoid the bias even if the participants might perceive their hydration state. After measuring the nude body weight, urine samples were collected.

### Interventions

At the second and third visits, participants came to the laboratory either in CON with adequate water intake or in MDEH with limited water intake for 24 h before the start of the experiment. This approach was chosen to minimize potential confounding factors, such as exercise, heat, and diuretic dehydration (Perry et al. 2016). Furthermore, to standardize the 24-h calorie intake between conditions, the participants were provided with packaged lunch and dinner meals and instructed to consume the same breakfast and snacks for each condition. Prior to each laboratory visit, the participants were required to complete 24-h diet sheets.

### Cognitive tasks

The participants performed two cognitive tasks with different difficulty levels designed to assess executive function before and after the 60-min exercise test. Go/No-Go and incongruent Stroop tasks were used as easy and difficult tasks, respectively.

In the Go/No-Go task, target stimuli (green circle) or non-target stimuli (red circle) were displayed for 1 s. When the target stimuli were displayed, the participants were instructed to press the space button on the computer with their right index finger as quickly as possible. If non-target stimuli were displayed, the participants were instructed to refrain from responding. This task consisted of 50 trials with equal probabilities (25 target stimuli and 25 non-target stimuli) (PsyToolkit version 3.4.0) (Stoet 2010, 2017). To evaluate the cognitive performance, the mean reaction time to the target stimuli and the accuracy of 50 trials in the Go/No-Go task were calculated.

In the incongruent Stroop task, colors words (red, blue, green, and yellow) were printed in different ink colors (red, blue, green, and yellow) displayed on a computer screen. To increase difficulty, all words presented were displayed in English for all participants whose native language was Japanese. The participants were instructed to press one of the four colored buttons with their right fingers on a computer keyboard (red, blue, green, or yellow) that corresponded to the real meaning of the word (red, blue, green, or yellow) presented on the computer screen as quickly as possible. The word presented and its ink color were randomly selected using a computer (100% incongruent). Each word was presented on a screen with a font size of 34 for 1500 ms, followed by a blank screen for 500 ms, before the next word was displayed (PsyToolkit version 3.4.0) (Stoet 2010, 2017). To evaluate the cognitive performance, the mean reaction time and accuracy of 50 trials in the incongruent Stroop task were calculated.

### Data acquisition

#### Heart rate and blood pressure

During the second and third visits, heart rate (HR) was continuously measured using a lead II electrocardiogram (BMS-3400; Nihon Kohden, Tokyo, Japan) at 1,000 Hz. Arterial blood pressure was measured via the right brachial artery using an upper-arm cuff device (EBP-330; Minato Medical Science Co.) to measure systolic and diastolic pressures. Arterial blood pressure was measured at each time point before evaluating the VAS.

#### Skin blood flow and sweat rate

Skin blood flow in the non-glabrous areas of the chest (SkBF_chest_) and forearm (SkBF_forearm_) was assessed using laser Doppler flowmetry (ALF21; Advance, Tokyo, Japan) at a sampling rate of 100 Hz. Local sweat rates at the chest (SR_chest_) and forearm (SR_forearm_) were assessed using dew hygrometry (SKD-4000; Skinos, Nagoya, Japan) at a sampling rate of 1 Hz. HR, SkBF, and SR were recorded using a 16-bit A/D converter (Power Lab 16 s; AD Instruments, Sydney, Australia), and data were stored on a personal computer.

#### Core body and skin surface temperatures

Ear canal temperature (T_ear_) was assessed as an index of core body temperature by infrared thermometry (VTB01; Vitarate, Tokyo, Japan) (Kato et al. 2023), which remained continuously inserted. The ear orifice was covered with medical film to reduce the direct influence of ambient ventilation. T_ear_ were collected on a smartphone (iPhone 11; Apple, Cupertino, USA) with an application program (Thermologger; Vitarate, Japan) every 30 s using Bluetooth. Skin surface temperatures of the anterior chest (T_chest_), upper arm (T_arm_), and lateral sides of the thigh (T_thigh_) and lower limb (T_limb_) were measured every 60 s using a temperature/humidity logger (iButton Hydrochron; Maxim, Dallas, USA) to estimate the mean skin surface temperature (mean T_skin_), calculated using the equation 0.3 × (T_chest_ + T_arm_) + 0.2 × (T_thigh_ + T_limb_) (Ramanathan 1964).

#### VAS and RPE

Thirst and thermal sensations, comfort of the whole body, humid sensation, and physical and psychological fatigue were self-recorded by the participants using VAS. The VAS consisted of a statement and a 200- or 100-mm straight line. One end of the line represented “extremely cold, uncomfortable, or dry” (− 100 mm) and the other end represented “extremely hot, comfortable, or humid” (100 mm) (Aizawa et al. 2019; Nagashima et al. 2022). For the assessment of thirst sensation and physical and psychological fatigue, one end of the line represented “no thirst or fatigue” (0 mm) and the other end represented “extremely thirst or completely exhausted” (100 mm) (Van Cutsem et al. 2017; Verschueren et al. 2020; Picó-Munyoz et al. 2023).

The Borg scale was used for determining RPE as follows: extremely light (6–8), very light (9–10), light (11–12), somewhat hard (13–14), hard (15–16), very hard (17–18), and extremely hard (19–20) (Borg 1982).

#### Data analyses

Mean arterial blood pressure (MAP) was calculated using the following formula: MAP = diastolic blood pressure + 1/3 × (systolic blood pressure − diastolic blood pressure). The absolute value of $\dot{V}$O_2_ for each participant was normalized by the nude body weight obtained on the first day and expressed as the percentage value of $\dot{V}$O_2peak_ (%$\dot{V}$O_2peak_). Cutaneous vascular conductance (CVC) was estimated using SkBF/MAP. SkBF, CVC, and SR were expressed as the difference (Δ) from each baseline value. HR, ΔLDF, ΔCVC, ΔSR, T_ear_, mean T_skin_, and %$\dot{V}$O_2peak_ were averaged over 2 min before the end of 30-min rest as baseline values and over 1 min before the end of exercise at each set as the 1st, 2nd, and 3rd set values.

**Fig. 2:**
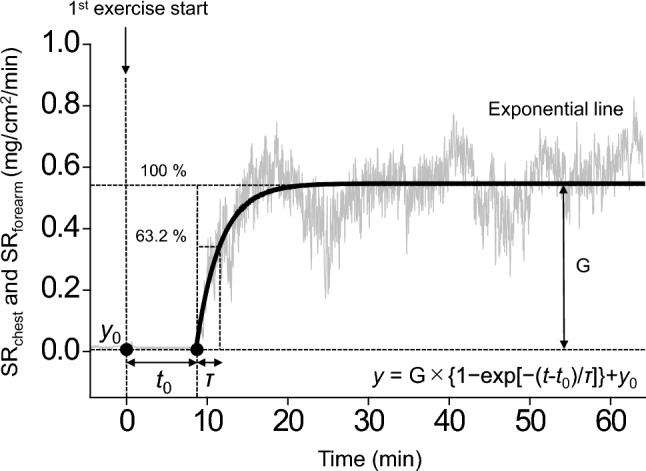
Single-exponential regression model; *t*_0_, response time latency from 1st exercise to change (sweat rate [SR]-response delay); *y*_0_, baseline value; G, gain term; and τ, time constant of the fitted curve of exponential regression during exercise protocol. Time 0 (*t*_0_ = 0) indicates start of the 1st exercise. *τ* is the time (unit: second) from *t*_0_ to reach 63.2% of the steady-state value

To estimate sweat response to exercise onset (i.e., non-thermal stimulus) (Kondo et al. 2010; Shibasaki and Crandall 2010), dynamic responses of SR in the chest and forearms from the start of 1st exercise were evaluated using a one-compartment non-linear least-squares optimization method. SR data were fitted to the following single-exponential regression equation consisting of the response-time latency [SR-response delay (*t*_0_)], baseline value, gain term (G), and time constant (*τ*) fitted to the exercise: *y* = G × {1 − exp[− (*t* − *t*_0_)/*τ*]} + *y*_0_, where *y* is the response, *t* is time, and *y*_0_ is the baseline value. Time zero was the start of 1st exercise (Fig. 2).

### Statistical analyses

Data are expressed as the mean ± standard deviation. Paired *t*-test was used to compare 24-h water and calorie intake between the CON and MDEH. Two-way analysis of variance (ANOVA) with repeated measures (condition × time) was used to analyze nude body weight, USG, %$\dot{V}$O_2peak_, HR, MAP, ΔSkBF, ΔCVC, ΔSR, T_ear_, mean T_skin_, VAS, RPE, corrected answer rate, and response time of the cognitive task. When a significant interaction or main effect was detected, post-hoc multiple comparisons were performed to identify pairwise differences using paired *t*-tests with Bonferroni correction. Statistical analyses were conducted using SPSS software (SPSS Statistics 27; IBM Corp., Armonk, NY, USA). Statistical significance was set at *P* < 0.05. The effect sizes were calculated as eta squared (*η*^2^) for one-way ANOVA outcomes and Cohen’s *d* values for paired *t*-test (Lakens 2013). Conservative interpretation for effect size were defined as small (*η*^2^ = 0.01, Cohen’s *d* = 0.20), medium (*η*^2^ = 0.06, Cohen’s *d* = 0.50), and large (*η*^2^ = 0.14, Cohen’s *d* = 0.80) effects (Lakens 2013). The statistical results, except for the *P* values, are presented in figures or tables. Variables not included in figures and tables are described in the text.

## Results

### Hydration state

The amount of water intake in 24 h was lower in MDEH (568 ± 131 mL) than that in CON (2883 ± 918 mL) (*t*_11_ = 8.943, *P* < 0.001, Cohen’s *d* = 2.584), whereas no significant difference in 24-h calorie intake was observed between the conditions (CON: 1787 ± 456 kcal and MDEH: 1794 ± 514 kcal) (*t*_11_ =  − 0.176, *P* = 0.863, Cohen’s *d* = 0.309). Consequently, ANOVA showed a main effect of condition and time for body weight and USG (Table 1). Post-hoc analysis revealed that body weight was lower in MDEH than that in CON (*P* = 0.007), whereas USG was higher in MDEH than that in CON (*P* < 0.001). Moreover, significant effects and interaction were observed for thirst sensation (Table 1). Post-hoc analysis revealed that thirst sensation was higher in MDEH than that in CON before and after the trials (both *P* < 0.005), although thirst sensation increased in both conditions from before to after the trial (both *P* < 0.001).

**Table 1:** Hydrated state in euhydrated (CON) and sustained mild dehydrated (MDEH) conditions

Variables	Condition	Before trial	After trial	Two-way ANOVA			
					Condition	Time	Interaction
Body weight (kg)	CON	58.44 ± 9.54	57.87 ± 9.37	*df*	1, 11	1, 11	1, 11
	MDEH	57.80 ± 9.38	57.20 ± 9.21	*F*	10.704	78.069	1.733
				*P*	0.007	< 0.001	0.215
				*η*^*2*^	0.001	0.272	< 0.001
USG	CON	1.008 ± 0.006	1.011 ± 0.005	*df*	1, 11	1, 11	1, 11
	MDEH	1.026 ± 0.004	1.025 ± 0.008	*F*	87.915	0.553	0.873
				*P*	< 0.001	0.473	0.370
				*η*^*2*^	0.634	0.004	0.011
Thirst sensation	CON	26 ± 17	60 ± 22^*^	*df*	1, 11	1, 11	1, 11
	MDEH	62 ± 21^†^	78 ± 15^*†^	*F*	23.832	39.210	5.062
				*P*	< 0.001	< 0.001	0.046
				*η*^*2*^	0.261	0.300	0.035

Data are shown as mean ± standard deviation

*USG* urine specific gravity

*P < 0.05 compared with the data obtained before the trial

^†^*P* < 0.05 compared with CON

### $\dot{V}$***O***_***2peak***_***, HR, and MAP***

Only significant effect of time was observed, without significant effects of condition and interaction for %$\dot{V}$O_2peak_ (time: *F*_1.507, 16.528_ = 327.240, *P* < 0.001, *η*^2^ = 0.958; condition: *F*_1, 11_ = 2.059, *P* = 0.179, *η*^2^ = 0.001; interaction: *F*_2.268, 24.951_ = 0.884, *P* = 0.437, *η*^2^ < 0.001). Post-hoc analysis revealed that %$\dot{V}$O_2peak_ increased from baseline in the 1st exercise set (*P* < 0.001), then remained stable (all *P* ≥ 0.856), and returned to the baseline value during recovery (*P* = 0.293).

**Fig. 3:**
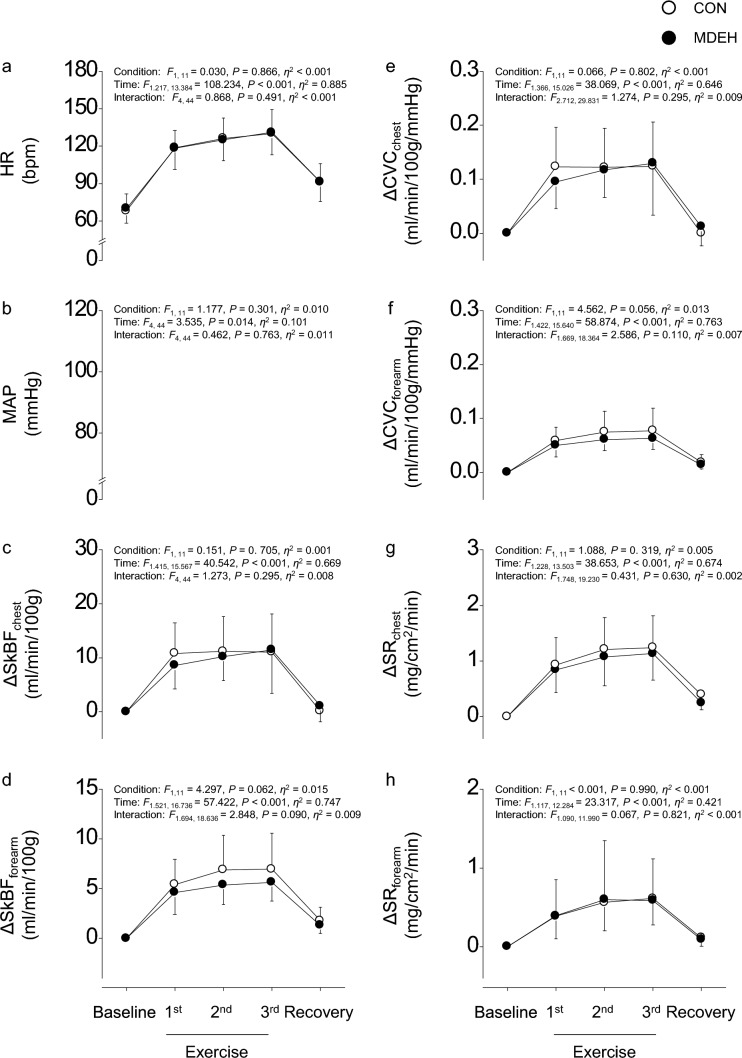
Group-averaged data. Heart rate (HR, **a**); mean arterial blood pressure (MAP, **b**); difference in skin blood flow for the chest (SkBF_chest_, **c**) and forearm (SkBF_forearm_, **d**); difference in cutaneous vascular conductance of the chest (CVC_chest_, **e**) and forearm (CVC_forearm_, **f**); and difference in sweat rate (SR_chest_, **g**) and (SR_forearm_, **h**) of the chest and forearm, respectively, during euhydrated (CON, white circle) and sustained mildly dehydrated (MDEH, black circle) conditions. Data are shown as mean ± standard deviation

Similar to %$\dot{V}$O_2peak_, a significant effect of time was observed without significant effects of condition and interaction for HR (Fig. 3a) and MAP (Fig. 3b). Post-hoc analysis revealed that HR increased consistently from baseline to 3rd exercise set (all *P* < 0.009) and decreased during recovery but remained higher than the baseline value (*P* = 0.001). In contrast, MAP was maintained throughout the trials (all *P* ≥ 0.098).

### SkBF, CVC, and SR

A significant effect of time was observed without significant effects of condition and interaction for ΔSkBF (Fig. 3c and Fig. 3d) and ΔCVC in the chest and arm (Fig. 3e and Fig. 3f). In the chest, post-hoc analysis revealed that ΔSkBF_chest_ and ΔCVC_chest_ increased from baseline to 1st exercise set (both *P* < 0.001), remained stable (all *P* ≥ 0.055), and returned to baseline value during recovery (both *P* = 1.000). In the forearm, ΔSkBF_forearm_ and ΔCVC_forearm_ increased from baseline to 1st exercise set (both *P* < 0.001), remained stable (all *P* ≥ 0.078), and decreased during recovery but remained higher than baseline (both *P* ≤ 0.003).

Similar to observations for SkBF, a significant effect of time was observed without significant effects of condition and interaction for ΔSR in the chest and forearm (Fig. 3g and Fig. 3h). In the chest, ΔSR_chest_ also increased from baseline to 2nd exercise set (all *P* ≤ 0.001), remained stable (all *P* = 1.000), and decreased during recovery but remained higher than baseline (*P* = 0.002). In the forearm, ΔSR_forearm_ increased from baseline to 2nd exercise set (all *P* ≤ 0.017), remained stable (*P* = 1.000), and decreased during recovery but remained higher than baseline (*P* = 0.005).

Moreover, the dynamic sweating response of *t*_0_, *y*_0_, G, *τ*, and *t*_0_ + *τ* in the chest and forearm were not different between CON and MDEH (Tables 2 and 3).

**Table 2:** Dynamic responses of sweat rate (SR) of the chest from the start of 1st exercise

Variables	Condition		*t*-test			
	CON	MDEH	*df*	*t*	*P*	Cohen’s *d*
*t*_0_, s	277 ± 272	248 ± 223	11	0.482	0.639	0.139
*y*_0_, mg/cm^2^/min	0.12 ± 0.12	0.11 ± 0.20	11	0.329	0.748	0.095
G	1.04 ± 0.62	1.21 ± 0.51	11	− 1.016	0.331	− 0.293
τ, s	677 ± 304	763 ± 451	11	− 0.766	0.460	− 0.221
*t*_0_ + τ, s	955 ± 378	1011 ± 395	11	− 0.404	0.694	− 0.117

Data are shown as mean ± standard deviation

*SR* response delay (*t*_0_), response time latency, *y*_*0*_ baseline value, *G* gain term, *τ* time constant, *CON* euhydrated condition, *MDEH* sustained mild dehydrated condition

**Table 3:** Dynamic responses of sweat rate (SR) of the forearm from the start of 1st exercise

Variables	Condition		*t*-test			
	CON	MDEH	*df*	*t*	*P*	Cohen’s *d*
*t*_0_, s	324 ± 271	319 ± 225	11	0.058	0.954	0.017
*y*_0_, mg/cm^2^/min	0.04 ± 0.05	0.05 ± 0.06	11	− 0.556	0.589	− 0.160
G	0.59 ± 0.31	0.57 ± 0.54	11	0.096	0.925	0.028
τ, s	875 ± 386	1069 ± 658	11	− 1.268	0.231	− 0.336
*t*_0_ + τ, s	1198 ± 437	1388 ± 576	11	− 1.014	0.332	− 0.293

Data are shown as mean ± standard deviation

*SR* response delay (*t*_0_), response time latency, *y*_*0*_ baseline value, *G* gain term, *τ* time constant, *CON* euhydrated condition, *MDEH* sustained mild dehydrated condition

### ***T***_***ear***_*** and mean T***_***skin***_

**Fig. 4:**
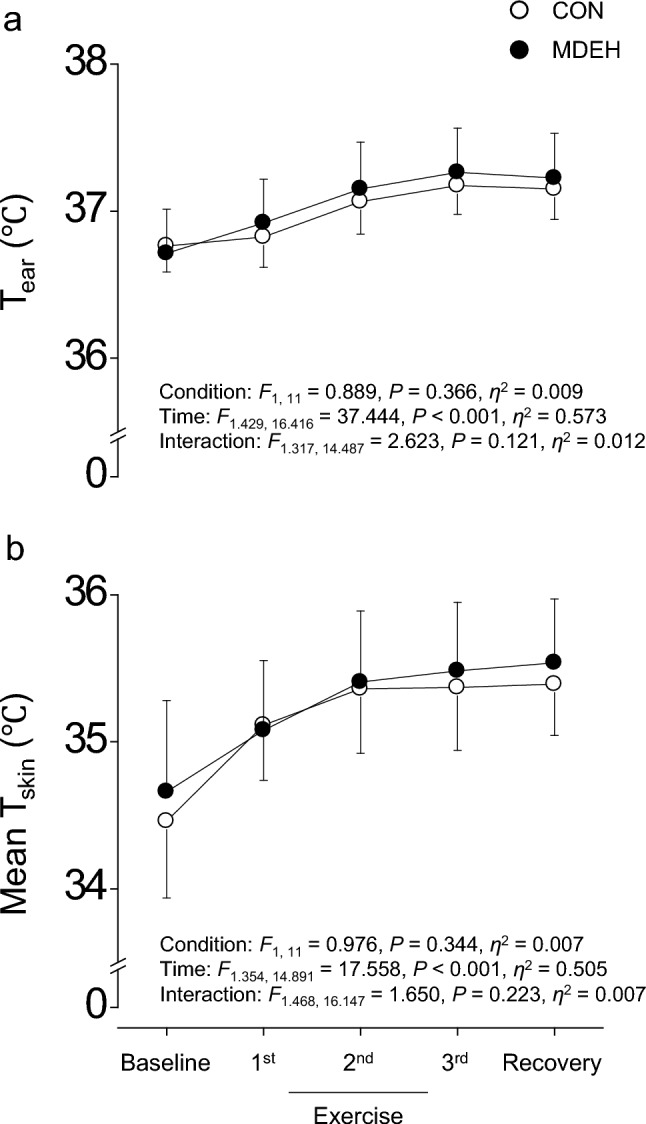
Group-averaged data of the ear canal (T_ear_, **a**) and mean skin surface temperature (mean T_skin_, **b**) during euhydrated (CON, white circle) and sustained mildly dehydrated (MDEH, black circle) conditions. Data are shown as the mean ± standard deviation

Similar to the responses of HR, MAP, SkBF, and SR, only significant effect of time was observed without significant effects of condition and interaction for T_ear_ (Fig. 4a) and mean T_skin_ (Fig. 4b). Post-hoc analysis revealed that T_ear_ and mean T_skin_ increased from baseline to 3rd (all *P* < 0.004) and 2nd exercise sets (all *P* < 0.028), respectively, and remained stable until the end of recovery (all *P* ≥ 0.268).

### VAS and RPE

**Fig. 5:**
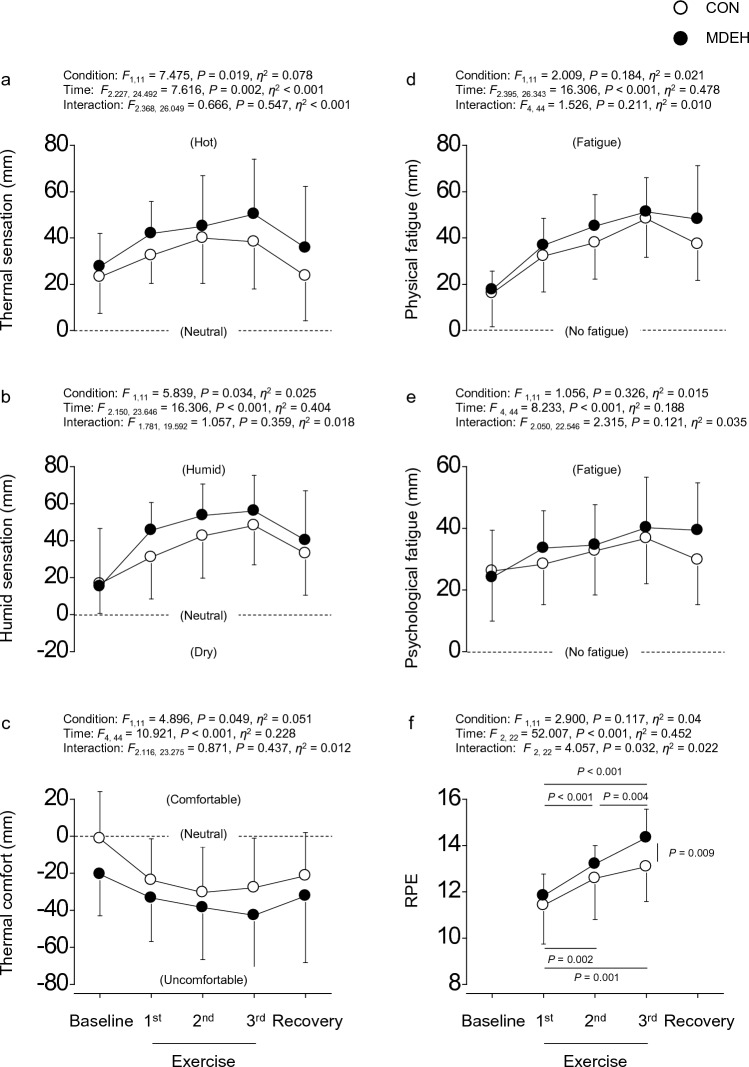
Group-averaged data of the visual analog scale (VAS)-assessed thermal (**a**) and humid sensations (**b**), thermal comfort (**c**), physical (**d**) and psychological fatigue (**e**), and the rating of perceived exertion (RPE) (**f**) during euhydrated (CON, white circle) and sustained mild dehydrated (MDEH, black circle) conditions. Data are shown as mean ± standard deviation

Regarding VAS, significant effects were observed, except for interaction, for thermal sensation (Fig. 5a), humid sensation (Fig. 5b), and thermal comfort (Fig. 5c). Post-hoc analysis revealed that thermal and humidity sensations were higher in MDEH than those in CON (both *P* < 0.034), whereas thermal comfort was lower in MDEH than that in CON (*P* = 0.049). Moreover, thermal and humid sensations increased from baseline to 1st exercise set (all *P* < 0.030), remained stable (all *P* ≥ 0.081), and returned to baseline during recovery (all *P* ≥ 0.081).

However, only significant effect of time was observed without significant effects of condition and interaction for physical fatigue (Fig. 5d) and psychological fatigue (Fig. 5e). Moreover, physical fatigue increased from baseline to 3rd exercise set (all *P* ≤ 0.003) and remained higher than baseline during recovery (*P* = 0.002), while mental fatigue increased at 3rd exercise set (*P* = 0.017) and remained higher than baseline during recovery (*P* = 0.033).

Regarding RPE, a significant interaction was observed (Fig. 5f). Post-hoc analysis revealed that RPE in MDEH increased from 1st to 3rd exercise sets (all *P* ≤ 0.004), whereas RPE in CON increased from 1st to 2nd exercise sets (*P* < 0.001) and remained steady (*P* = 0.158). Moreover, RPE in MDEH was higher than that in CON for 3rd exercise set (*P* = 0.009).

### Cognitive performance

Regarding Go/No-Go task, no significant effects and interaction were observed for the correct answer rate and response time (Table 4).

**Table 4:** Cognitive performance in the euhydrated (CON) and sustained mild dehydrated (MDEH) conditions

Variables	Condition	Before trial	After trial	Two-way ANOVA			
					Condition	Time	Interaction
Go/No-Go task							
Correct answer rate (%)	CON	99.2 ± 1.3	99.8 ± 0.6	*df*	1, 11	1, 11	1,11
	MDEH	99.3 ± 1.8	99.2 ± 1.6	*F*	1.000	0.292	1.536
				*P*	0.339	0.600	0.241
				*η*^*2*^	0.008	0.014	0.038
Response time (ms)	CON	353.91 ± 28.36	344.25 ± 42.48	*df*	1, 11	1, 11	1, 11
	MDEH	346.6 ± 37.78	349.16 ± 38.37	*F*	0.042	0.288	0.576
				*P*	0.841	0.602	0.464
				*η*^*2*^	< 0.001	0.008	0.023
Incongruent Stroop task							
Correct answer rate (%)	CON	97.7 ± 3.4	97.5 ± 2.3	*df*	1, 11	1, 11	1, 11
	MDEH	97.8 ± 2.6	98.5 ± 1.9	*F*	1.803	0.128	0.589
				*P*	0.206	0.727	0.459
				*η*^*2*^	0.013	0.006	0.015
Response time (ms)	CON	619.42 ± 44.94	619.79 ± 59.39	*df*	1, 11	1, 11	1, 11
	MDEH	654.68 ± 66.97^†^	624.15 ± 64.68^*^	*F*	4.069	5.495	5.468
				*P*	0.069	0.039	0.039
				*η*^*2*^	0.028	0.080	0.084

Data are shown as mean ± standard deviation

*P < 0.05 compared with data obtained before the trial

^†^*P* < 0.05 compared with CON

In contrast, a significant interaction was observed for the response time of the incongruent Stroop task, whereas no significant interaction and effects were observed for corrected answer rate (Table 4). Post-hoc analysis revealed that before exercise sets, response time was slower in MDEH by 5.62 ± 6.37% than that in CON (*P* = 0.011). However, after exercise sets, the response time in MDEH shortened (*P* = 0.011) and was not different from that in CON (*P* = 0.724).

## Discussion

This study demonstrated four important findings. First, physiological responses to prolonged moderate exercise in warm environments were comparable in both CON and MDEH, and no differences in T_ear_ and mean T_skin_ between the two conditions were observed. Second, despite the comparable physiological responses, thermal perception of the environment was augmented. Third, the perceived exercise strength increased in MDEH. Fourth, MDEH impaired cognitive performance in tasks with greater difficulty.

We did not assess the change in body mass and plasma osmolality across 24 h to directly estimate actual water loss due to overnight restriction of water intake. Instead, we assessed the loss based on the difference in body weight between the two conditions. Because caloric intake was adjusted between the two conditions, the influence of metabolism and metabolic water production on body weight would have been low. Lower body weight in MDEH than in CON (1.08 ± 1.15%; Table 1) with USG of > 1.025 (1.026 ± 0.004) suggested mild dehydration in MDEH, as expected (Perry et al. 2016). Despite the reduction in body fluid in MDEH, HR and MAP, reflecting systemic circulatory response, were not different between the two conditions (Fig. 3). Moreover, $\dot{V}$O_2_ at the given treadmill speed was not influenced by the dehydration state, suggesting that dehydration did not affect the relative workload for each participant. A previous study showed that moderate-to-severe dehydration was accompanied by a decrease in blood volume, which increased HR to maintain arterial pressure via the cardiopulmonary baroreflex (Crandall and González-Alonso 2010). These results suggest that the circulatory blood volume was maintained in MDEH at the same level as that in CON. This inference is supported by interventions similar to those in this study, which induced comparable levels of mild dehydration assessed by USG (~ 1.025); however, plasma osmolality was negligible (~ 290 mosmol/kg) (Perry et al. 2016). One possible reason for this is fluid movement from the intracellular to the extracellular space by an osmotic drive (Nose et al. 1988). The higher thirst rating in MDEH suggests fluid movement (Thornton 2010). Therefore, although we did not measure the body mass and plasma osmolality across 24 h, the circulatory blood volume could be maintained in MDEH.

Isotonic hypovolemia inhibits thermoregulatory cutaneous vasodilation while maintaining the sweat response to heat (Ikegawa et al. 2011; Takamata 2012). However, SkBF, CVC, and SR were similar between CON and MDEH (Fig. 3), suggesting that autonomic thermoregulatory function was not influenced by sustained mild dehydration in this study. Moreover, similar values of T_ear_ and mean T_skin_ under the two conditions (Fig. 4) may indicate that thermal inputs/outputs from the central and peripheral nervous systems are not influenced by sustained mild dehydration.

We assessed the dynamic response of sweating at the onset of 1st exercise to evaluate the non-thermal factors stimulating sweating (Shibasaki and Crandall 2010) (Fig. 2 and Tables 2 and 3). Although SR can increase without elevating the core body or skin surface temperature (Van Beaumont and Bullard 1966; Kondo et al. 1999), the onset of exercise-induced SR response is a multifaceted process influenced by various mechanisms. These include the activation of mechanosensitive (Van Beaumont and Bullard 1966) and metabosensitive receptors (Kondo et al. 1999) in exercising muscles, central command signals linked to voluntary effort (Van Beaumont and Bullard 1966; Kondo et al. 1997), and processes related to emotional or mental stimuli (Ogawa 1975). In this study, the same exercise load was performed by both CON and MDEH with no change in $\dot{V}$O_2_, indicating equivalent activation of mechanosensitive or metabosensitive receptors. However, we hypothesized that if the central command signals, as well as emotional or mental stimuli, were enhanced in the MDEH, the onset of sweating and time to reach a steady state would be faster under the MDEH. Despite these hypotheses, no significant differences in dynamic response of SR were observed between CON and MDEH (Fig. 2 and Tables 2 and 3), although the subjective thermal perceptions and RPE were augmented under the MDEH. This implies that MDEH influences factors related to the onset of sweating but not to a greater extent.

An important finding of this study was that hot and humid sensations and thermal discomfort increased with exercise. Moreover, these changes were augmented in MDEH compared with those in CON (Fig. 5a, c). In humans, thermal discomfort is a strong factor initiating thermoregulatory behavior (Nagashima et al. 2018). Therefore, sustained mild dehydration may be a stimulus facilitating the behavior.

In warm or hot environments, thermal discomfort increases with both hot and humid sensations (Berglund 1997, 1998; Jing et al. 2013; Amaripadath et al. 2023). Both the core body and skin surface temperatures per se are factors that change thermal sensation (Xu and Lian 2024). Moreover, sweating changes thermal sensation via evaporation from the skin and increases thermal discomfort via mechanical stimulation of the skin (Berglund 1997). However, there were no differences in T_ear_, mean T_skin_, and ∆SR between CON and MDEH (Fig. 3 and 4). Therefore, the reason for the increase in the humid sensation and thermal discomfort in MDEH remains unclear. One plausible explanation is that there may be an interaction between thermal perception and thirst sensation. We previously identified the brain regions involved in thermal sensation (medial prefrontal cortex/anterior cingulate gyrus, inferior frontal gyrus, bilateral insula, and posterior parietal cortex) and those involved in thermal comfort and discomfort (medial prefrontal cortex, posterior cingulate cortex, and inferior parietal lobes) (Aizawa et al. 2019; Nagashima et al. 2022). The anterior cingulate cortex and insula are activated during thirst (Gizowski and Bourque 2017). Therefore, the simultaneous activation of sensations involved in heat and thirst may augment thermal discomfort during exercise in sustained mild dehydration.

RPE, an index of the level of activation of the central command (Franke et al. 2000; Williamson et al. 2002), was higher in MDEH than that in CON (Fig. 5f). The exact underlying mechanism is unclear in this study; however, thirst sensation may be involved. Negative psychological associations attributable to thirst may act as a signaling mechanism to promote a higherer conscious perception of effort, thus invoking a behavioral change to reduce physical effort (Edwards et al. 2007). However, exercise may be maintained but only at the expense of a higher metabolic cost, as observed by increased neural activity of the frontoparietal brain region during a cognitive task (Kempton et al. 2011). Therefore, MDEH may negatively affect motivation and increase effort perception, resulting in reduced central motor drive during exercise. On the contrary, dehydration also affects peripheral muscle contractile function, resulting in increased brain activity even under the same workload, but this phenomenon occurs under moderate or severe dehydrated states (Uddin et al. 2022). Therefore, it is plausible that increased RPE is associated with motivation and effort due to thirst itself.

It is widely acknowledged that MDEH impairs cognitive functions, including execution, attention, perception, and psychomotor functions (Ganio et al. 2011). In this study, compared to CON, the response time in the incongruent Stroop task before the exercise trial was higher in MDEH, whereas no corresponding changes were observed in the Go/No-Go task (Table 4). This implies that the negative impact of MDEH on cognitive function is contingent on task difficulty. Initially, we considered that cognitive impairment leads to attenuated behavioral responses, thereby increasing the risk of heat stroke. Contrary to this expectation, thermal perception-related behavioral responses became more sensitive under the MDEH rather than less responsive. Furthermore, the impaired response time in Stroop task improved after exercise. Extensive evidence has established that acute exercise enhances cognitive functions (Ando et al. 2020). In addition, even under condition of moderate dehydration, cognitive performance can be maintained by exercise (Ando et al. 2023), suggesting that the potential benefits of exercise may outweigh the drawbacks of MDEH and improve cognitive function. Thus, this study could not definitively establish a correlation between cognitive function and thermal perceptions. On the other hand, the negative impact of the MDEH on cognitive function is contingent on task difficulty. The difference between cognitive tasks used experimentally and those encountered in real-world scenarios needs to be considered; however, real-world work demands often entail tasks with higher cognitive requirements, and sports activities frequently require a high degree of precision. In situations where cognitive performance is compromised, the risk of severe accidents and subpar performance are likely to increase. Therefore, this study serves as a cautionary note regarding these risks.

The present study has some limitations. The results of this study cannot be generalized to the elderly or patients with impaired thermoregulation because all participants were healthy young adults. Moreover, all participants did not acclimate and acclimatize to the heat. Since participants’ aerobic fitness levels were classified as performance level 2, the results cannot be extended to the individuals of all fitness levels due to the varying impacts of mild dehydration on the physiological and psychological effects observed across different levels of fitness (Merry et al. 2010). Furthermore, the exercise protocol used in this study consisted of a 1-h session at 40% $\dot{V}$O_2peak_, and the results may vary depending on the workload and exercise style. In addition, the environmental conditions used in this study (30 °C and 60% RH) may not be representative of other settings. Given that this study was conducted at the laboratory level, it remains uncertain whether these results would be replicated in real-world settings.

In conclusion, by controlling 24-h water intake, we assessed the effect of sustained mild dehydration on physiological and psychological responses during prolonged exercise in a warm environment. We found that autonomic thermoregulatory function was well-maintained. However, sustained mild dehydration augmented thermal perception, including hot and humid sensations and thermal discomfort. Perceived exertion was increased by dehydration. Dehydration impairs cognitive performance during difficult tasks. We may not have been able to detect the influence of sustained mild dehydration during exercise by evaluating physiological variables.

## Data Availability

All data generated or analysed during this study are included in this published article.

## Abbreviations

CON: Euhydrated condition
CVC: Cutaneous vascular conductance
HR: Heart rate
MAP: Mean arterial pressure
MDEH: Mild dehydrated condition
SkBF: Skin blood flow
RPE: Rating of perceived exertion
SR: Sweat rate
T_ear_: Ear canal temperature
T_skin_: Skin surface temperature
USG: Urine specific gravity
VAS: Visual analog scale
$$\dot{V}$$O_2_: Oxygen uptake
WBGT: Wet-bulb globe temperature
