# Correction to: Influence of sustained mild dehydration on thermoregulatory and cognitive functions during prolonged moderate exercise

**DOI:** 10.1007/s00421-024-05602-3

**Published:** 2024-10-09

**Authors:** Hironori Watanabe, Yuma Kadokura, Taisuke Sugi, Kiyoshi Saito, Kei Nagashima

**Affiliations:** 1 Institute for Energy and Environmental System, Sustainable Energy & Environmental Society Open Innovation Research Organization, Waseda University, 3-4-1 Okubo, Shinjuku-ku, Tokyo, 1698555 Japan; 2 Advanced Research Center for Human Sciences, Waseda University, 2-579-15 Mikajima, Tokorozawa, Saitama 3591192 Japan; 3 Body Temperature and Fluid Laboratory, Faculty of Human Sciences, Waseda University, 2-579-15 Mikajima, Tokorozawa, Saitama 3591192 Japan; 4 Infrastructure Systems Research & Development Center, Toshiba Infrastructure Systems & Solutions Corporation, Kawasaki, Kanagawa 2129595 Japan; 5 Department of Applied Mechanics and Aerospace Engineering, School of Fundamental Science and Engineering, Waseda University, 3-4-1 Okubo, Shinjuku-ku, Tokyo, 1698555 Japan

**Correction to: European Journal of Applied Physiology** 10.1007/s00421-024-05548-6

In the original version of this article, data for Fig. 3b (MAP) was not presented. The Fig. 3 should have appeared as shown below.

**Fig. 3:**
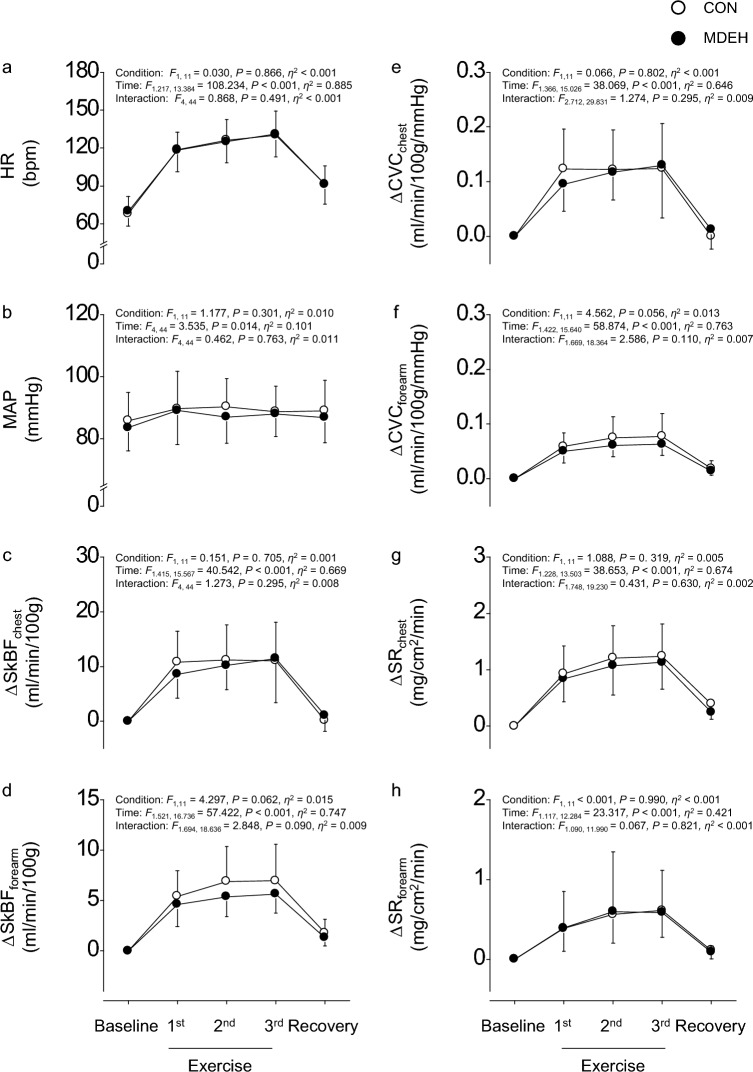
Group-averaged data. Heart rate (HR, **a**); mean arterial blood pressure (MAP, **b**); difference in skin blood flow for the chest (SkBF_chest_, **c**) and forearm (SkBF_forearm_, **d**); difference in cutaneous vascular conductance of the chest (CVC_chest_, **e**) and forearm (CVC_forearm_, **f**); and difference in sweat rate (SR_chest_, **g**) and (SR_forearm_, **h**) of the chest and forearm, respectively, during euhydrated (CON, white circle) and sustained mildly dehydrated (MDEH, black circle) conditions. Data are shown as mean ± standard deviation

